# High levels of serum β2-microglobulin predict severity of coronary artery disease

**DOI:** 10.1186/s12872-017-0502-9

**Published:** 2017-03-01

**Authors:** Ling You, Ruiqin Xie, Haijuan Hu, Guoqiang Gu, Hongmei Zheng, Jidong Zhang, Xiaohong Yang, Ximiao He, Wei Cui

**Affiliations:** 1 Division of Cardiology, The Second Hospital of Hebei Medical University, 215 Heping West Rd, Xinhua, Shijiazhuang, Hebei 050000 People’s Republic of China; 2 Laboratory of Metabolism, National Cancer Institute, National Institutes of Health, 37 Convent Drive, Bethesda, MD 20892 USA

**Keywords:** β2-Microglobulin, Severity, Coronary artery disease

## Abstract

**Background:**

The identification of new risk factors for coronary artery disease (CAD) is increasingly sought in an effort to tackle this threatening disease. β2-microglobulin (B2M) is reported to associate with peripheral arterial disease and adverse cardiovascular outcomes. However, the association between B2M and cardiovascular disease remains under-researched. This study evaluated the effects of B2M on CAD without renal dysfunction.

**Methods:**

One thousand seven hundred sixty-two subjects (403 non-CAD subjects and 1,359 CAD subjects) were investigated. Fasting samples were collected to determine B2M level. The Gensini and SYNTAX scores were used to assess the severity of CAD.

**Results:**

CAD subjects were significantly higher in serum B2M level comparing with non-CAD subjects (1.25 ± 0.46 vs 1.14 ± 0.28 mg/L, *p* < 0.001). Serum B2M level was a risk factor of CAD after adjusting potential confounders (Odds Ratio (OR) = 2.363, 95% confidence interval (CI): 1.467–3.906, *p* = 0.001). Receiver operating characteristics (ROC) showed B2M level moderately predicted diagnosis of CAD (the area under the ROC curve (AUC) = 0.608, 95% CI: 0.577–0.639, *p* < 0.001). Furthermore, serum B2M level was positively associated with Gensini score system, SYNTAX score system and the number of disease vessels (NDV ≥ 2).

**Conclusions:**

The significant association between serum B2M and CAD suggests that B2M could be a biomarker for CAD.

**Electronic supplementary material:**

The online version of this article (doi:10.1186/s12872-017-0502-9) contains supplementary material, which is available to authorized users.

## Background

Coronary artery disease (CAD) has remained the first death burden globally between 2000 and 2012. It was responsible for over 7.4 million deaths in 2012. Although, both genders of all ages may develop CAD, males and the elderly are more vulnerable to death causing by CAD [1, 2]. Quality of life was also remarkably reduced in CAD patients. Nearly a third patients had angina attacks at least once per week [3]. Therefore, there is imperative focus to identify new CAD risk factors. Several biomarkers, such as C-reactive protein [4, 5], natriuretic peptides [6, 7] and sensitive cardiac troponins [8, 9] have been used to predict risk of CAD.

β2-microglobulin (B2M), a low molecular-weight protein (~11,800 Da), is a component of the major histocompatibility complex (MHC) class I molecules on all nucleated cells [10]. Due to the rapid, simple, reliable, and inexpensive measurement of B2M, it is commonly used by clinicians to evaluate conditions such as: dialysis-related amyloidosis [11], human immunodeficiency virus (HIV) disease [12], myeloma [13], leukemia [14], and collagen disease [15]. B2M has been correlated to inflammatory diseases [16] and is also an estimator of the glomerular filtration rate (GFR) [17].

Recent studies have shown that circulating B2M is elevated in accordance with the acuteness of disease in peripheral arterial disease (PAD) patients [18–20]. High levels of serum B2M was also associated with adverse cardiovascular outcomes in patients with CAD [21, 22]. Aysegul Zumrutdal has reported B2M was positively correlated with the carotid intima-media thickness (C-IMT) in haemodialysis patients [23]. However, there is no confirming evidence of the relationship between serum B2M and the severity of CAD. In this study, we examined the relationship between the concentrations of serum B2M and severity of CAD.

## Methods

### Study population

Two thousand two-hundred consecutive subjects admitted to a university hospital with the suspected or already documented of CAD (included acute coronary syndrome and stable angina), who underwent selective coronary angiography (CAG) between June 2011 and July 2012, participated as the candidates for study. A self-administered questionnaire was conducted (covering items on demographic, disease history, cigarette and alcohol consumption (defined as one or more alcoholic drink per week)). Diabetes mellitus history was defined as a fasting plasma glucose level > 126 mg/dL, or 2-h post-load blood sugar > 200 mg/dL, or glycated hemoglobin (A1c) ≥ 6.5%, or using anti-diabetic drugs. Hypertension was defined as systolic blood pressure (SBP) ≥ 140 mmHg or diastolic blood pressure (DBP) ≥90 mmHg or taking anti-hypertensive medications.

A total of 390 subjects were excluded where blood samples were not measured, or a questionnaire was not completed. 1,810 subjects with serum β2-microglobulin and creatinine levels were selected as candidates for further study. Among them, 48 subjects with creatinine levels of ≥ 115 umol/L were removed to exclude the subjects with possibility of renal dysfunction. Finally, 1,762 subjects were included in this study, with 403 non-CAD subjects (referred to those with normal coronary angiography or vessel stenosis <50%), and 1,359 CAD subjects (those with positive coronary artery angiography and vessel stenosis ≥ 50%). This observational study complied with the tenets of the Declaration of Helsinki and was approved by Clinical Research Ethical Committee of Hebei Medical University. All informed consents were signed by participants before conducting.

### Laboratory analysis

In all cases, blood samples were drawn following a minimum 12 h overnight fast. All the tests were performed using standard biochemical techniques to determine the following parameters: β2-microglobulin (B2M, mg/L), triglyceride (mmol/L), total cholesterol (mmol/L), high-density lipoprotein cholesterol (HDL-cholesterol, mmol/L), low-density lipoprotein cholesterol (LDL-cholesterol, mmol/L), apolipoprotein-A (g/L), apolipoprotein-B (g/L), fasting blood glucose (mmol/L), creatinine (umol/L), blood urea nitrogen (BUN, mmol/L), and uric acid (UA, umol/L).

### Coronary angiography

Two experienced interventional cardiologists (M.D. and M.G.K.) measured quantitative coronary angiography using the standard Judkins approach. Both had no knowledge of subjects’ clinical information. Two or more clinicians cross-checked the CAG reports and determined the degree of coronary stenosis according to the American Heart Association standards [24]. Coronary stenosis was determined to be significant at ≥50%. Left anterior descending arteries, left circumflex arteries, and right coronary arteries were measured to determine the number and range of stenotic coronary arteries (SCA) (0 to 3-vessel disease (VD)). 2-VD was independently recorded when coronary stenosis was found in the left main trunk. Stenotic arteries was counted using the scoring systems, Gensini [25] and SYNTAX [26], to determine the extent of CAD.

### Statistical analysis

All subjects were categorized into 4 groups according to B2M quartile ranges. Data was presented as the mean ± SD (standard deviation) for scaled measurements or percentages for categorized values. Analysis of variance (ANOVA) was performed for testing continuous data. Kruskal-Wallis test was used for not normally distributed outcomes. Categorical comparison was tested through Chi-square test. Pearson correlations and Spearman correlations were performed to determine statistically significant factors into multiple regression model. Biologically relevant factors were also selected into multiple regression model to exam the association between B2M and CAD as well as B2M and Gensini score or SYNTAX score. Receiver operating characteristics (ROC) curve was used to assess the prediction accuracy of CAD by B2M. Statistically significance was set as *P* < 0.05. All the statistical analyses were conducted by Statistical Package for Social Sciences (SPSS) software (version 12 for Windows, SPSS, Inc., Chicago, IL, USA).

## Results

### Characteristics of the study subjects

**Fig. 1:**
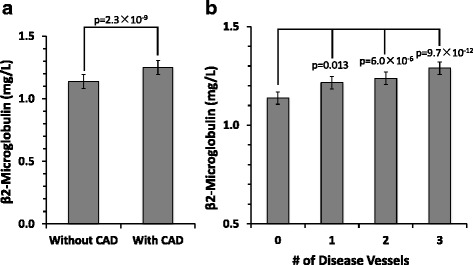
B2M levels between patients with CAD (*n =* 1,359) and those without CAD (*n =* 403). **a** Comparison of serum B2M levels in non-CAD vs CAD. **b** Comparison of serum B2M levels according to the number of stenotic coronary arteries (NVD = 0, 1, 2, and 3)

One thousand seven hundred sixty-two subjects were eligible for the study (403 non-CAD subjects and 1,359 CAD subjects). CAD subjects were elder than non-CAD subject (58.60 ± 9.59 vs 55.65 ± 9.68, *p <* 0.001). More males were observed in the CAD subjects (70.6% vs 47.8%, *p <* 0.001). The prevalence of smoking, drinking, hypertension, diabetes mellitus, and acute myocardial infarction (AMI) in CAD subjects was higher than non-CAD subjects (Table 1). CAD subjects also had a higher value of creatinine concentrations, blood urea nitrogen (BUN), uric acid (UA), triglyceride, apolipoprotein-A and apolipoprotein-B. B2M levels were significantly lower in non-CAD subjects (1.14 ± 0.28 vs 1.25 ± 0.46 mg/L, *p <* 0.001; Fig. 1a).

**Table 1:** Clinical and biochemical characteristics of the study subjects

	Non-CAD (*n =* 403)	CAD (*n =* 1,359)	*p-*value
Age (years)	55.65 ± 9.68	58.60 ± 9.59	<0.001
Gender (male, %)	47.8	70.6	<0.001
Smoking (%)	18.9	39.4	<0.001
Drinking (%)	15.9	25.8	<0.001
Hypertension (%)	47.8	63.5	<0.001
Diabetes (%)	9.2	19.4	<0.001
AMI (%)	1.2	7.6	<0.001
HF (%)	0.5	0.7	0.992
SBP	131.54 ± 18.56	133.44 ± 21.22	0.108
DBP	81.29 ± 12.08	81.15 ± 20.89	0.378
HR (bpm)	68.04 ± 14.68	70.49 ± 13.08	0.008
Creatinine (umol/L)	66.58 ± 15.25	71.2 ± 15.3	<0.001
BUN (mmol/L)	5.25 ± 4.2	6.38 ± 18.96	<0.001
UA (umol/L)	285.44 ± 137.57	306.84 ± 179.28	<0.001
Glu	7.44 ± 31.96	6.48 ± 18.86	<0.001
TG (mmol/L)	1.6 ± 1.01	2 ± 5.19	0.002
TC (mmol/L)	4.36 ± 1.83	4.67 ± 11.29	0.424
HDL-C (mmol/L)	1.49 ± 5.22	1.09 ± 0.45	0.000
LDL-C (mmol/L)	2.58 ± 0.77	2.95 ± 9.76	0.055
Apo-A (g/L)	1.37 ± 0.27	1.3 ± 0.71	<0.001
Apo-B (g/L)	0.88 ± 0.25	1.15 ± 4.9	<0.001
B2M (mg/L)	1.14 ± 0.28	1.25 ± 0.46	<0.001
Gensini score	0.5 ± 1.69	39.34 ± 34.66	<0.001

*CAD* coronary artery disease, *AMI* acute myocardial infarction, *HF* heart failure, *SBP* systolic blood pressure, *DBP* diastolic blood pressure, *HR* heart rate, *BUN* blood urea nitrogen, *UA* uric acid, *Glu* blood glucose, *TG* Triglycerides, *TC* total cholesterol, *HDL-C* high-density lipoprotein cholesterol, *LDL-C* low-density lipoprotein cholesterol, *Apo-A* Apolipoprotein A, *Apo-B* Apolipoprotein B, *B2M* β2-microglobulin. Data are means ± SD

All 1,762 subjects were also categorized into 4 groups based on B2M quartile ranges (Additional file 1: Figure S1): 1st quartile (<1.00 mg/L), 2nd quartile (1.00-1.17 mg/L), 3rd quartile (1.18-1.35 mg/L), and 4th quartile (>1.35 mg/L). To eliminate the possibility of renal dysfunction, subjects only in the normal range of creatinine were included (Additional file 2: Figure S2). As shown in Table 2, we compared the main baseline demographic, clinical and angiographic characteristics between the four quartiles. There was an increasing in B2M level with aging (*p <* 0.001). Significant measurements were also included drinking, hypertension, diabetes mellitus, myocardial infarction, baseline apolipoprotein-A, HDL-C, creatinine, uric acid (UA) and blood urea nitrogen (BUN) (Table 2).

**Table 2:** Baseline clinical and angiographic features based on quartiles of B2M

Characteristics	Quartile 1	Quartile 2	Quartile 3	Quartile 4	*p-*value
	≤1.00 (mg/L) *n* = 472	1.00–1.17(mg/L) *n* = 443	1.18–1.35(mg/L) *n* = 431	>1.35(mg/L) *n* = 416	
Baseline clinical features					
Age (years)	53.85 ± 9.44	56.49 ± 9.12	60.38 ± 8.53	61.58 ± 9.55	<0.001
Gender (male, %)	63.0	68.7	65.8	63.9	0.285
Smoker (%)	33.3	38.3	34.9	32.0	0.242
Drinker (%)	25.5	28.0	21.7	18.3	0.005
Hypertension (%)	52.9	59.0	61.3	67.7	<0.001
Diabetes (%)	15.2	16.0	15.5	22.1	0.022
AMI (%)	3.6	5.9	8.5	6.9	0.018
HF (%)	0.4	0.5	0.9	0.7	0.751
Baseline blood features					
TG (mmol/L)	1.86 ± 1.33	2.01 ± 5.09	2.12 ± 7.51	1.64 ± 1.03	0.228
TC (mmol/L)	4.39 ± 0.99	4.42 ± 1.01	4.39 ± 1.88	5.26 ± 20.39	0.044
HDL-C (mmol/L)	1.14 ± 0.49	1.38 ± 4.98	1.1 ± 0.33	1.09 ± 0.58	0.000
LDL-C (mmol/L)	2.65 ± 0.82	2.74 ± 0.92	2.62 ± 0.85	3.49 ± 17.6	0.295
Apo-A (g/L)	1.31 ± 0.29	1.41 ± 1.15	1.27 ± 0.26	1.25 ± 0.38	<0.001
Apo-B (g/L)	1.25 ± 6.59	1.19 ± 5.19	0.94 ± 0.4	0.94 ± 0.36	0.730
Creatinine (umol/L)	64.13 ± 13.68	68.48 ± 13.12	71.36 ± 15.56	77.4 ± 16.13	<0.001
BUN (mmol/L)	5.78 ± 14.45	5.15 ± 1.26	6.89 ± 19.98	6.74 ± 23.13	<0.001
UA (umol/L)	272.97 ± 76.96	299.38 ± 88.34	300.06 ± 120.89	338.75 ± 301.73	<0.001
Angiographic features					
CAD (%)	68.1	75.7	79.1	86.1	<0.001
Severe CAD^a^ (%)	29.4	46.5	52.2	82.2	<0.001
Number of stenotic arteries					<0.001
0 (%)	37.2	27.1	22.3	21.7	
1 (%)	26.6	28.0	25.6	21.3	
2 (%)	22.1	25.2	26.2	24.5	
3 (%)	14.1	19.8	25.9	32.6	
Gensini score	24.94 ± 31.95	28.31 ± 34.42	30.02 ± 32.54	39.5 ± 37.73	<0.001

^a^Severe CAD defined as the patients with number of stenotic arteries ≥2. Data are means ± SD

*CAD* coronary artery disease, *AMI* acute myocardial infarction, *HF* heart failure, *SBP* systolic blood pressure, *DBP*, diastolic blood pressure, *HR* heart rate, *BUN* blood urea nitrogen, *UA* uric acid, *TG* Triglycerides, *TC* total cholesterol, *HDL-C* high-density lipoprotein cholesterol, *LDL-C* low-density lipoprotein cholesterol, *Apo-A* Apolipoprotein A, *Apo-B* Apolipoprotein B, *B2M* β2-microglobulin

### Relationship between serum B2M and prevalence of CAD

In univariate analysis, age, gender, smoking, drinking, hypertension, diabetes mellitus and AMI were statistically associated with CAD. Further, clinical predictors (heart failure, hypertension and heart rate (HR)) and biochemical risk factors (creatinine, BUN, UA, TG, TC, HDL-C, LDL-C, apolipoprotein-A, apolipoprotein-B, and B2M) were involved into multiple logistic regression model. After adjusted all the confounders, B2M (odds ratio, OR = 2.363, 95% confidence interval (CI): 1.467–3.906, *p =* 0.001), gender (OR = 2.247, CI: 1.668–3.041, *p <* 0.001), age (OR = 1.035, CI: 1.021–1.050, *p <* 0.001), smoking (OR = 1.865, CI: 1.336–2.62, *p <* 0.001), hypertension (OR = 1.382, CI: 1.074–1.792, *p =* 0.014), diabetes (OR = 2.278, CI: 1.532–3.541, *p <* 0.001), AMI (OR = 6.224, CI: 2.272–25.708, *p =* 0.02) and HDL-C (OR = 0.626, CI: 0.479–0.806, *p <* 0.001) were predictors of CAD (Table 3).

**Table 3:** Multiple stepwise regression analysis showing variables independently associated with CAD

	Odds Ratio (OR)	95% CI	*p-*value
B2M	2.363	1.467–3.906	0.001
Gender	2.247	1.668–3.041	<0.001
Age	1.035	1.021–1.050	<0.001
Smoking	1.865	1.336–2.620	<0.001
Hypertension	1.382	1.074–1.792	0.014
Diabetes	2.278	1.523–3.514	<0.001
AMI	6.224	2.272–25.708	0.002
HDL-C	0.626	0.479–0.806	<0.001

*CAD* coronary artery disease, *B2M* β2-microglobulin, *AMI* acute myocardial infarction, *HDL-C* high-density lipoprotein cholesterol

**Fig. 2:**
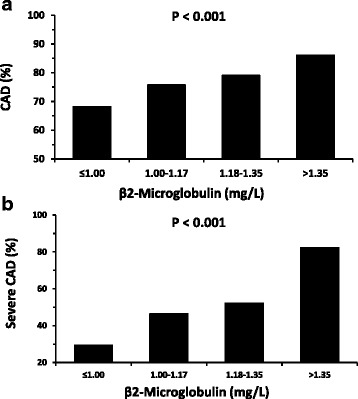
Bar graphs show the prevalence of CAD (**a**) and severe CAD (**b**) according to B2M quartiles

The correlation of B2M levels and prevalence of CAD was further confirmed in all four quartiles of B2M levels (CAD prevalence in B2M quartiles: 68.1% vs 75.7% vs 79.1% vs 86.1%, OR (95%CI) =1.432 (1.288–1.602), *p <* 0.001, Fig. 2a, Table 2). The positive trend was also observed with increased number of stenotic arteries (*p <* 0.001, Table 2, Additional file 3: Figure S3).

**Fig. 3:**
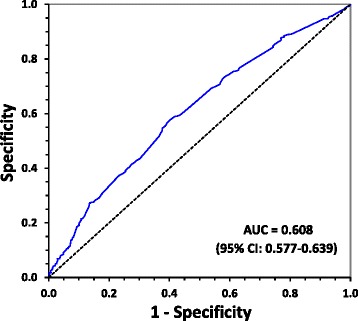
ROC curve analyses for predictive values of B2M in detecting CAD

The ability of correctly diagnosing CAD and non-CAD subject according to B2M was assessed using the receiver operating characteristics (ROC) curve. B2M showed a moderate predictive ability for CAD according to the area under the ROC curve (AUC = 0.608,95% CI: 0.577–0.639, *p =* 0.001) (Fig. 3).

### Relationship between serum B2M and clinical or biochemical parameters

B2M was positively correlated with age (Pearson Correlation Coefficient (r_p_) r_p_ = 0.207, *p <* 0.001), SBP (r_p_ = 0.073, *p* = 0.004), creatinine (r_p_ = 0.203, *p <* 0.001) and UA (r_p_ = 0.113, *p <* 0.001) (Table 4). In multiple regression, age (standardized β = 0.197, *p <* 0.001), SBP (standardized β = 0.057, *p* = 0.020), creatinine (standardized β = 0.179, *p <* 0.001), and UA (standardized β = 0.095, *p <* 0.001) were still associated with B2M.

**Table 4:** Correlation of serum B2M with clinical and biochemical parameters

Variables	r	*p-*value
Age	0.207	<0.001
SBP	0.073	0.004
DBP	0.005	0.833
HR	0.021	0.407
Creatinine	0.203	0.000
BUN	0.029	0.262
UA	0.113	0.000
Glu	0.004	0.877
TG	−0.014	0.572
TC	0.033	0.195
HDL-C	−0.016	0.519
LDL-C	0.010	0.683
Apo-A	−0.037	0.149
Apo-B	−0.008	0.749

*SBP* systolic blood pressure, *DBP* diastolic blood pressure, *HR* heart rate, *BUN* blood urea nitrogen, *UA* uric acid, *Glu* blood glucose, *TG* Triglycerides, *TC* total cholesterol, *HDL-C* high-density lipoprotein cholesterol, *LDL-C* low-density lipoprotein cholesterol, *Apo-A* Apolipoprotein A, *Apo-B* Apolipoprotein B

### Relationship between serum B2M and severity of CAD

**Fig. 4:**
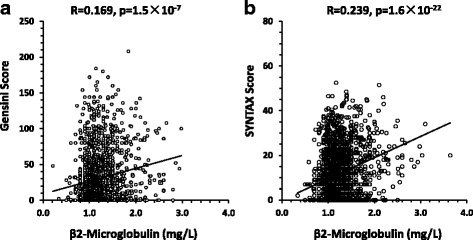
Correlation between B2M and the severity of CAD evaluated by either (**a**) Gensini or (**b**) SYNTAX score

B2M was positively correlated with Gensini score (Spearman Correlation Coefficient (r_s_) r_s_ = 0.169, *p <* 0.001; Fig. 4a). We further assessed the relationship by a multiple stepwise regression model adjusting age, gender, smoking, hypertension, diabetes mellitus and AMI. After adjustment, serum B2M was still significantly associated with the Gensini score (β = 0.078, 95% CI: 0.029–0.127, *p =* 0.002; Table 5).

**Table 5:** Multiple stepwise regression analysis showing variables independently associated with Gensini score

Variables	Coefficients (β)	95% CI	*p-*value
B2M	0.078	0.029–0.127	0.0019
Gender	0.132	0.077–0.188	<0.001
Age	0.107	0.058–0.157	<0.001
Smoking	0.070	0.015–0.126	0.0125
Hypertension	0.052	0.004–0.101	0.0333
AMI	0.124	0.076–0.172	<0.001
Diabetes	0.105	0.057–0.153	<0.001

*B2M* β2-microglobulin, *AMI* acute myocardial infarction

We also performed the analysis in SYNTAX score. B2M was correlated with SYNTAX score (r_s_ = 0.239, *p <* 0.001, Fig. 4b). In multiple stepwise regression, SYNTAX score was increased 0.077 as B2M level increased 1 mg/L (β = 0.077, 95% CI: 0.029–0.126, *p =* 0.002; Table 6).

**Table 6:** Multiple stepwise regression analysis showing variables independently associated with Syntax score

Variables	Coefficients (β)	95% CI	*p-*value
B2M	0.077	0.029–0.126	0.0018
Gender	0.160	0.105–0.215	<0.001
Age	0.154	0.104–0.203	<0.001
Smoking	0.049	−0.006–0.103	0.0802
Hypertension	0.062	0.014–0.109	0.0114
AMI	0.149	0.101–0.196	<0.001
Diabetes	0.109	0.061–0.156	<0.001

*B2M* β2-microglobulin, *AMI* acute myocardial infarction

Proportion of severe CAD was increased with higher B2M levels (29.4% vs 46.5% vs 52.2% vs 82.2%, *p <* 0.001, Fig. 2b, Table 2). B2M increased with the number of stenotic vessels with the lowest level in NVD of 0 (1.14 ± 0.28, 1.22 ± 0.61, 1.24 ± 0.32, 1.29 ± 0.39 mg/L in NVD of 0, 1, 2, and 3, respectively (Fig. 1b)).

## Discussion

Coronary artery disease (CAD) is associated with a high rate of mortality. Scientists and researchers have attempted to reduce the burden by identifying the relationship between biomarkers and CAD. In recent years, several biomarkers - C-reactive protein [4, 5], natriuretic peptides [6, 7] and sensitive cardiac troponins [8, 9] - have been used to estimate the risk of CAD. Some researchers have also investigated the relationship between coagulation factors (such as Fibrinogen, Thrombin, and Tissue Factor), development of atherosclerosis and thrombotic complication [27, 28], so as to optimize the treatment of CAD patients.

This is the first study to reveal the effects of B2M plasmatic level in the extent of coronary atherosclerosis in a large consecutive Chinese population. Our study demonstrated the B2M was not only associated with CAD prevalence, but also positively correlated with severity of CAD. One study has reported the correlation between B2M and carotid atherosclerosis severity in renal failure patients [23]. However, biased study population and limited the sample size constrained the conclusion to extend to other population.

The protein B2M is a non-glycosylated polypeptide consisted of 99 amino acids, and it can interact with and stabilize the tertiary structure of the MHC I α-chain [29]. B2M is not directly attached to cell membranes, due to its non-covalently association with the α-chain. After released inside the cell or detached from cell surfaces, B2M is then largely removed via glomerular filtration. It is this process that allows the glomerular filtration rate to be estimated [30]. In comparison to healthy subjects (serum B2M <2ug/mL, and urinary excretion <400ug/24 h [29]), patients on dialysis have greatly elevated B2M levels which contribute to amyloid deposition and cardiovascular dysfunction [31]. Autoimmune, neoplastic, and infectious diseases, such as multiple myeloma, lymphoma, and Sjogren’s syndrome have also reported to associate with increased plasma levels of B2M [32–34].

Although the mechanism for the association between B2M levels and CAD remains to be clarified, the relationship between B2M and alterations in vascular structures, immunity and inflammation disorders, suggests B2M may contribute to vascular inflammation [35, 36]. Atherosclerotic syndromes are also predominantly associated with an inflammatory response [37, 38], which leads the relationship between B2M and CAD. Furthermore, studies have shown that B2M concentrations were significant non-renal predictors of cardiovascular outcomes, renal outcomes, and mortality [39–43].

Researchers have provided association between B2M and CAD risk factors [22, 35], and left atrial size [44]. Liu YS found a positive relationship of B2M to serum levels of creatinine and a negative relationship to creatinine clearance rate [44]. To further build on these studies, we divided the participants into four quartiles according to the serum B2M levels, as well as the number of SCA (0 to 3-VD). We observed the B2M level was remarkably associated with the prevalence of severe CAD (29.4% for B2M ≤1 mg/L, while 82.2% for B2M >1.35 mg/L). Another important observation was that the B2M level was positively correlated with creatinine, especially in the subjects with severe CAD. Liu YS also found a linear trend between uric acid and B2M, indicating a potential link between the kidney and the heart [44].

There were some limitations in this study. First, we did not have a control group for the CAD or suspected diagnosis of CAD. Although the participants with number of SCA of 0 were treated as control group (non-CAD), all subjects in our study were consecutive at least with the suspected diagnosis of CAD. Therefore, the control group participants may be with a high risk for CAD. Second, this is an observational study, the follow up outcomes of B2M levels on cardiovascular was unavailable.

Our study reveals a strong association between level of serum B2M and CAD (both prevalence and severity) in subjects without renal dysfunction. These findings provide support to the potential of B2M as a biomarker for CAD. Further studies are need to ensure the potential benefits of B2M level for CAD in clinical routine.

## Conclusions

The significant association between serum B2M and CAD suggests that B2M could be a biomarker for CAD.

## Additional files

Additional file 1: Figure S1. The distribution of concentrations of B2M in all 1,762 subjects. (PPT 91 kb)
Additional file 2: Figure S2. The distribution of creatinine levels in all 1,762 subjects. (PPT 88 kb)
Additional file 3: Figure S3. The number of stenotic arteries in each quartile by B2M levels. (PPT 79 kb)
