# Supplementary material for: High levels of serum β2-microglobulin predict severity of coronary artery disease
Source: BMC Cardiovasc Disord. 2017 Mar 1;17:71. doi: 10.1186/s12872-017-0502-9 (PMC5333396; doi:10.1186/s12872-017-0502-9)
Supplement: Additional file 1: Figure S1. — The distribution of concentrations of B2M in all 1,762 subjects. (PPT 91 kb) [file 12872_2017_502_MOESM1_ESM.ppt]

## Slide 1
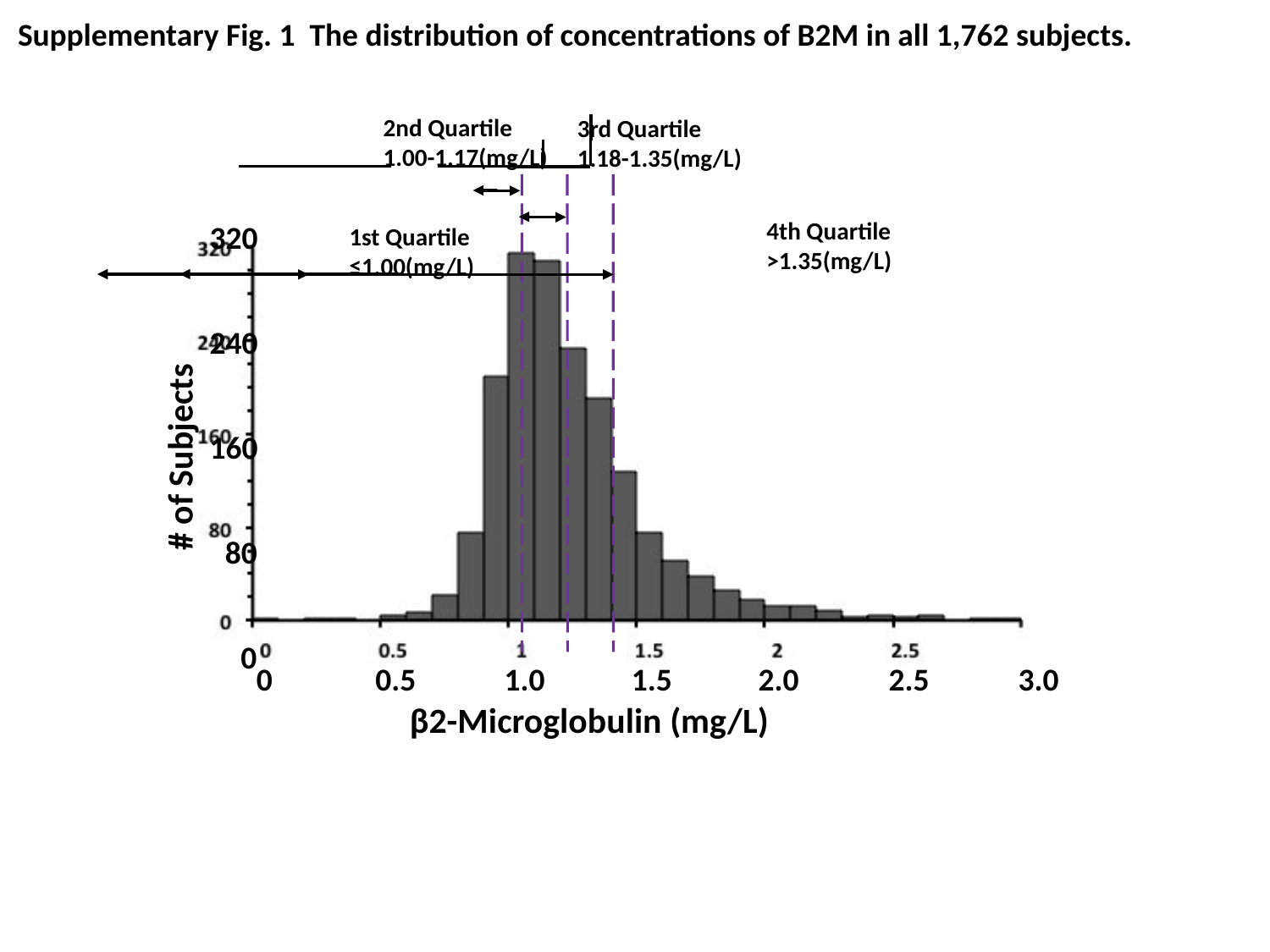

Supplementary Fig. 1 The distribution of concentrations of B2M in all 1,762 subjects.
2nd Quartile
1.00-1.17(mg/L)
3rd Quartile
1.18-1.35(mg/L)
4th Quartile
>1.35(mg/L)
320
240
160
80
0
0
0.5
1.0
1.5
2.0
2.5
3.0
1st Quartile
≤1.00(mg/L)
# of Subjects
β2-Microglobulin (mg/L)
