# Supplementary material for: High levels of serum β2-microglobulin predict severity of coronary artery disease
Source: BMC Cardiovasc Disord. 2017 Mar 1;17:71. doi: 10.1186/s12872-017-0502-9 (PMC5333396; doi:10.1186/s12872-017-0502-9)
Supplement: Additional file 2: Figure S2. — The distribution of creatinine levels in all 1,762 subjects. (PPT 88 kb) [file 12872_2017_502_MOESM2_ESM.ppt]

## Slide 1
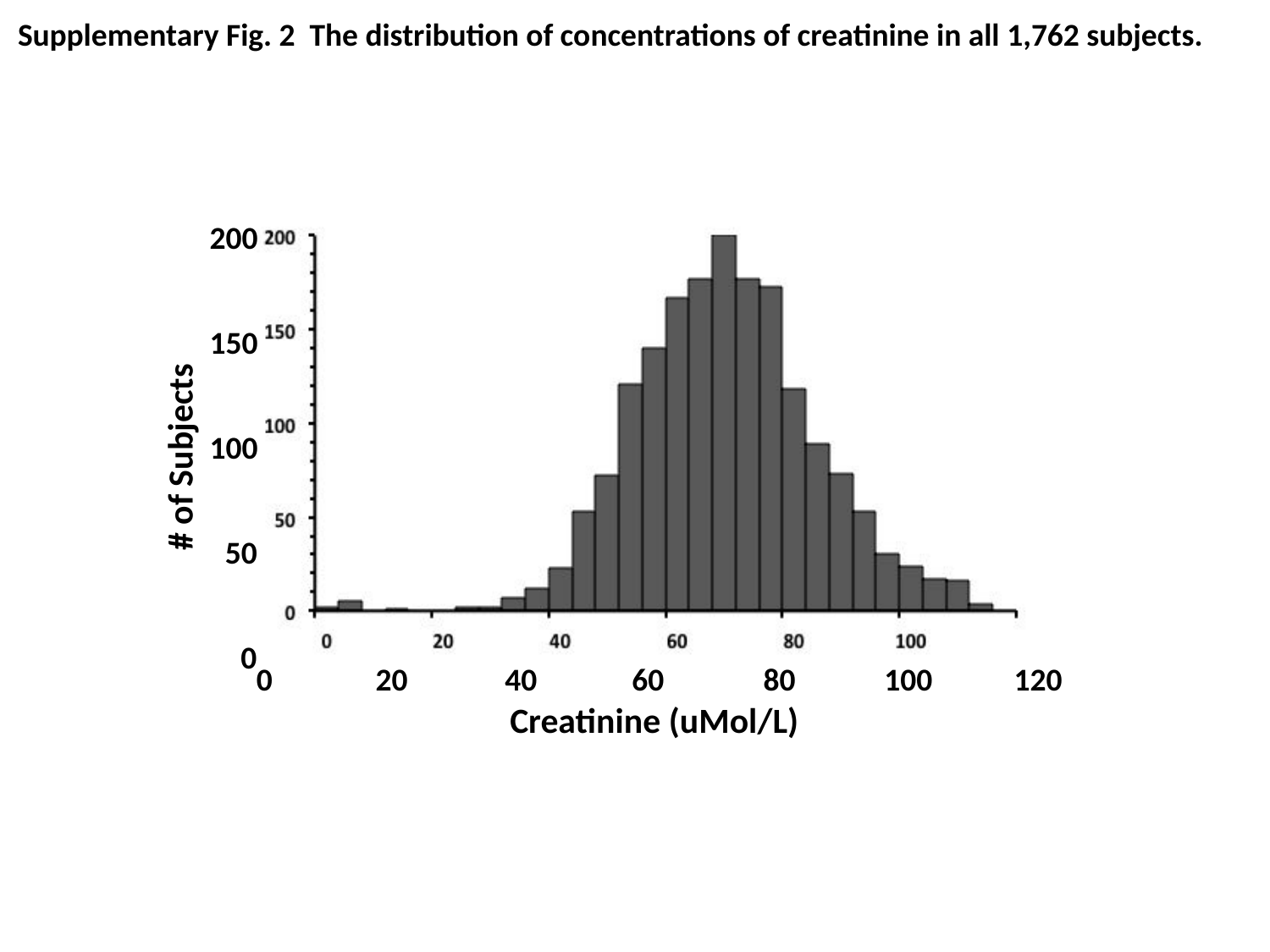

Supplementary Fig. 2 The distribution of concentrations of creatinine in all 1,762 subjects.
200
150
100
# of Subjects
50
0
0
20
40
60
80
100
120
Creatinine (uMol/L)
