# Supplementary material for: High levels of serum β2-microglobulin predict severity of coronary artery disease
Source: BMC Cardiovasc Disord. 2017 Mar 1;17:71. doi: 10.1186/s12872-017-0502-9 (PMC5333396; doi:10.1186/s12872-017-0502-9)
Supplement: Additional file 3: Figure S3. — The number of stenotic arteries in each quartile by B2M levels. (PPT 79 kb) [file 12872_2017_502_MOESM3_ESM.ppt]

## Slide 1
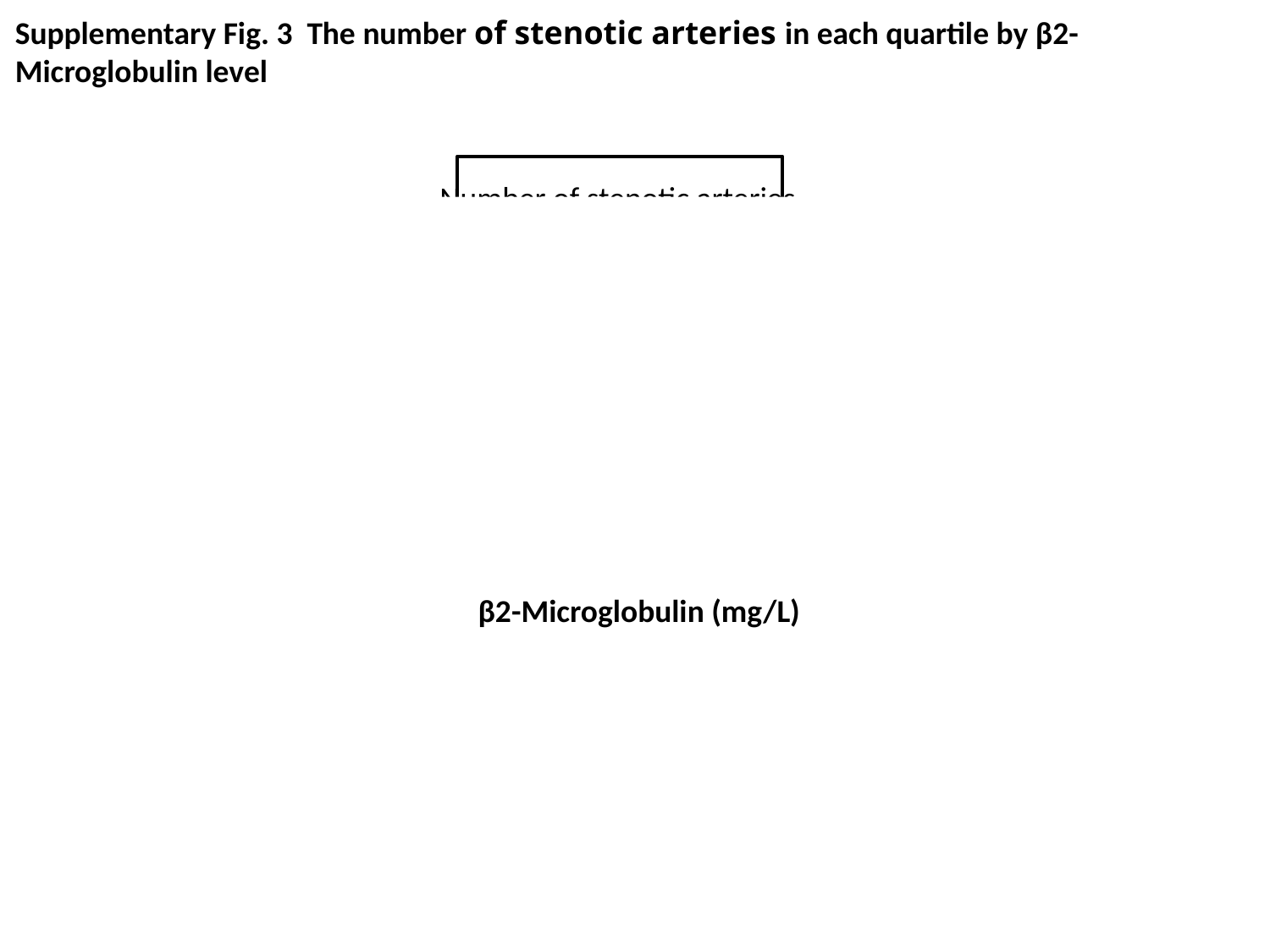

Supplementary Fig. 3 The number of stenotic arteries in each quartile by β2-Microglobulin level
Number of stenotic arteries
In each quartile (%)
β2-Microglobulin (mg/L)
### Chart
| Category | | | | |
|---|---|---|---|---|P < 0.001
